# Municipal Meat-inspection

**Published:** 1898-01

**Authors:** 


### MUNICIPAL MEAT-INSPECTION.

PHILADELPHIA'S initial meeting, in December, for better municipal meat-inspection, was a very important gathering, addressed by some of the ablest writers and exponents of a pure-food supply in our land. This meeting under the auspices of the Woman's Health Protective Association, now pledged to the accomplishment of this reform, will be followed by others under the direction of several other organizations, whose interest has been aroused in this subject, and now, with the promised aid from a large body of producers, consumers, health-boards, health-protective associations, medical and veterinary organizations, it is confidently hoped that the reform will be early inaugurated. The entire press of the city, lay and medical, is giving the movement a most encouraging support.

---

